# Adaptive Immunity against *Leishmania* Nucleoside Hydrolase Maps Its C-Terminal Domain as the Target of the CD4+ T Cell–Driven Protective Response

**DOI:** 10.1371/journal.pntd.0000866

**Published:** 2010-11-09

**Authors:** Dirlei Nico, Carla Claser, Gulnara P. Borja-Cabrera, Luiz R. Travassos, Marcos Palatnik, Irene da Silva Soares, Mauricio Martins Rodrigues, Clarisa B. Palatnik-de-Sousa

**Affiliations:** 1 Departamento de Microbiologia Geral, Instituto de Microbiologia Prof. Paulo de Góes, Universidade Federal do Rio de Janeiro (UFRJ), Rio de Janeiro, Brazil; 2 Centro Interdisciplinar de Terapia Gênica, Universidade Federal de São Paulo (UNIFESP), São Paulo, Brazil; 3 Unidade de Oncologia Experimental, Universidade Federal de São Paulo, São Paulo, Brazil; 4 Hospital Universitário Clementino Fraga Filho-Faculdade de Medicina, Universidade Federal do Rio de Janeiro, Rio de Janeiro, Brazil; 5 Departamento de Análises Clínicas e Toxicológicas, Universidade de São Paulo, São Paulo, Brazil; Queensland Institute of Medical Research, Australia

## Abstract

Nucleoside hydrolases (NHs) show homology among parasite protozoa, fungi and bacteria. They are vital protagonists in the establishment of early infection and, therefore, are excellent candidates for the pathogen recognition by adaptive immune responses. Immune protection against NHs would prevent disease at the early infection of several pathogens. We have identified the domain of the NH of *L. donovani* (NH36) responsible for its immunogenicity and protective efficacy against murine visceral leishmaniasis (VL). Using recombinant generated peptides covering the whole NH36 sequence and saponin we demonstrate that protection against *L. chagasi* is related to its C-terminal domain (amino-acids 199–314) and is mediated mainly by a CD4+ T cell driven response with a lower contribution of CD8+ T cells. Immunization with this peptide exceeds in 36.73±12.33% the protective response induced by the cognate NH36 protein. Increases in IgM, IgG2a, IgG1 and IgG2b antibodies, CD4+ T cell proportions, IFN-γ secretion, ratios of IFN-γ/IL-10 producing CD4+ and CD8+ T cells and percents of antibody binding inhibition by synthetic predicted epitopes were detected in F3 vaccinated mice. The increases in DTH and in ratios of TNFα/IL-10 CD4+ producing cells were however the strong correlates of protection which was confirmed by *in vivo* depletion with monoclonal antibodies, algorithm predicted CD4 and CD8 epitopes and a pronounced decrease in parasite load (90.5–88.23%; p = 0.011) that was long-lasting. No decrease in parasite load was detected after vaccination with the N-domain of NH36, in spite of the induction of IFN-γ/IL-10 expression by CD4+ T cells after challenge. Both peptides reduced the size of footpad lesions, but only the C-domain reduced the parasite load of mice challenged with *L. amazonensis*. The identification of the target of the immune response to NH36 represents a basis for the rationale development of a bivalent vaccine against leishmaniasis and for multivalent vaccines against NHs-dependent pathogens.

## Author Summary

The continued spread, morbidity and mortality of human leishmaniasis together with the emergence of drug-resistant variants, the failure of epidemiological control based on dog culling and insecticide vector control and the chemotherapy toxicity have spurred attempts to develop an effective vaccine. Leishmaniasis affects 12 million people and another 350 million live under risk worldwide. We developed the first licensed second generation vaccine against leishmaniasis, a canine vaccine that has already reduced the incidence of the human and canine disease in endemic areas. Its main component is the Nucleoside hydrolase of *Leishmania donovani* (NH36) which in its recombinant and DNA formulation is cross protective against agents of tegumentary leishmaniasis (TL). For this work we generated three recombinant peptides covering the NH36 sequence and identified the C-domain of the Nucleoside hydrolase as being responsible for its immunogenicity and vaccine-induced protective efficacy against VL and also for the reduction of lesion size and parasite load against TL. Since all *Leishmanias* species share high identity in their Nucleoside hydrolases amino acid sequences, our study represents a major step forward in the development of a bivalent synthetic vaccine against leishmaniasis and a potential future multivalent vaccine against pathogens that are dependent on NHs for replication.

## Introduction

In recent years, Nucleoside hydrolases (NHs) of trypanosomatid protozoa have emerged as strong phylogenetic markers of the *Leishmania* genus [1], [2] and vital protagonists of pathways for parasite replication and establishment of infection. The purine-dependent protozoa: *Crithidia fasciculata* [3], *Trypanosoma brucei* [4], *Trypanosoma cruzi* [5], *Leishmania major* [6], *Leishmania donovani* [7], [8] and *Leishmania infantum* [2] like most protozoan parasites, are deficient in *de novo* synthesis of purines. NHs cleave the N-glycosidic linkage of imported nucleosides making the purines available for further parasite DNA synthesis. NHs activities have also been described in bacteria and fungi [9], [10], [11] but not in mammals [11], which have alternative pathways.

Since NHs are expressed in the early stages of infection, they are excellent candidate targets for pathogen recognition by adaptive immune responses. NHs of *Leishmania* have been described in the parasite stages which infect the mammal host [1],[2],[6],[7],[8] and in the exosporium membrane of *Bacillus anthracis* being important for anthrax transmission [10]. Vaccines against NHs would then prevent the replication of many different pathogens at the very first stage of their life-cycle and thus prevent infection, mild disease, severe disease and death while vaccine with antigens present in later stages of the parasite cycle would only protect from severe disease and death [12].

The NH of *L. donovani* shows significant homology to the sequences of *L. major* (95%) [7], *L. chagasi* (99%), *L. infantum* (99%), *L. tropica* (97%), *L. mexicana* (93%), *L. braziliensis* (84%) [13], *T. brucei* (27%) and *Crithidia fasciculat*a (80%) [7] and shares 68% identity with *Haemophylus influenzae* and 30% identity and conserved motifs with *Bacillus anthracis* [10], [13]. Identification of the immunogenic molecular domain of the NH of one pathogen should allow the rational design development of a cross-protective subunit or synthetic vaccine and this would explain the protection generated by NH of *Leishmania donovani* against infections by other leishmanias [14]–[17].

However, the role of the Nucleoside hydrolases in the induction of immunoprotective CD4+ T cell driven or CD8^+^ T cell-mediated cytotoxic immune response has never before been systematically examined in the context of parasitic diseases. We developed the first licensed second generation vaccine against visceral leishmaniasis [18]–[21] that has already reduced the incidence of the human and canine disease in endemic areas [22]. Its main component is the Nucleoside hydrolase of *Leishmania donovani* (NH36) which was specifically recognized by sera of patients of human VL [23] and by most anti-FML monoclonal antibodies [24]. According to the guidelines of WHO [12], NH36 was first identified as a powerful antigen present in the early stages of the parasite infection. Its NH nature and degree of identity to other *Leishmanias* NHs was only disclosed after molecular cloning [8]. In its native form it protected mice from infection by *L. donovani* [25] and was also identified by polyclonal antibodies among promastigote exo-antigens [7]. In its recombinant or DNA formulations it protected mice from infection by *L. chagasi*, *L. mexicana* [14], [15], *L. amazonensis* [16] and *L. major* [17] and dogs from infection by *L. chagasi* [26] indicating the potential use of its sequence in protection against both leishmaniasis. As a bivalent vaccine, it induced a TH1 immune response mediated by IFN-γ-producing CD4+ T cells which led to a 88% prophylaxis against visceral leishmaniasis (VL) [14], 65–81% against tegumentary leishmaniasis (TL) [14], [16], [17] and 91% immunotherapy against VL [27]. Also, higher proportions of CD4+-NH36 specific lymphocytes and higher levels of IFN-γ, IL-2 were found in NH36-vaccinated dogs than in untreated controls [26].

NH36 is composed of a 314 amino acid sequence [7]. In order to map the domain which is the target of the adaptive immunity, three recombinant fragment proteins representing the amino acids 1–103 (F1, N-terminal domain), 104–198 (F2, central domain) and 199–314 (F3, C-terminal domain) were generated and used for the stimulation of splenocytes of NH36 vaccinated mice, which secreted IFN-γ and TNF-α after stimulation with the F3 followed by the F1 fragment, confirming the induction of a cellular protective TH1 immune response [28].

An effective subunit vaccine against VL must include T cell epitopes capable of eliciting protective immune responses, since progressive suppression of the cellular immunity is one of the main signs of the disease. Synthetic vaccines based on short peptides which represent immunogenic epitopes are able to impair and even exceeded the protective potential of the native cognate whole protein [29] and they can also induce universal T cell responses, which are related to many human HLA-DR allotypes and to diverse mice genetic backgrounds [30], [31].

In this investigation, we vaccinated mice with the F1, F2 and F3 recombinant peptides and saponin in order to identify the NH36 protective epitopes recognized by antibodies and by MHC class I and II restricted T cells and then move on to develop a Nucleoside hydrolase based synthetic vaccine against VL. We identified the C-terminal domain of the Nucleoside hydrolase NH36 as being responsible for the adaptive immunity and vaccine-induced protective efficacy.

## Material and Methods

### Ethical statements

All mouse studies followed the guidelines set by the National Institutes of Health, USA and the Institutional Animal Care and Use Committee approved the animal protocols (Biophysics Institute-UFRJ, Brazil, protocol IMPPG-007).

### Recombinant peptides and epitopes of the NH36 Nucleoside Hydrolase

NH36 is composed of 314 aminoacids (EMBL, Genbank and DDJB data bases, access number AY007193). Three fragments of the NH36 antigen composed respectively, of the amino acid sequences 1–103 (F1), 104–198 (F2) and 199–314 (F3) were cloned in the pET28 plasmid system. Fragments were amplified by PCR with the Platinum Taq High Fidelity DNA polymerase (Invitrogen) and oligonucleotides containing the NcoI and XhoI restriction sites, cloned into the pMOS vector (GE) for sequencing confirmation and further cloned into pET 28b. The recombinant proteins were expressed in *E. coli* Bl21DE3 cells and purified in a Ni-NTA column (Qiagen). To improve protein expression, F2 was further cloned in the pET28a. The NH36 protein amino acid sequence was analyzed using epitope prediction algorithms based on MHC-binding motifs. Epitopes for antibodies and CD4+ lymphocytes were defined by the Protean Pad program based on the A. Sette algorithm for the H2^d^ haplotype of Balb/c mice (IA^d^ and IE^d^ alleles) and epitopes for CD8+ T cells (H2 L^d^ haplotype), by the HLA peptide motif search (http://bimas.dcrt.nih.gov/molbio/hla_bind/) and the SYFPEITHI (http://www.syfpeithi.de/) programs.

### Immunization and parasite challenge by *Leishmania chagasi*

Female Balb/c mice, 8-week-old, were vaccinated at weekly intervals, by the sc route, with 3 doses of 100 µg of NH36, F1, F2 or F3 recombinant proteins and 100 µg of SIGMA saponin [32]. On week 4, mice were challenged with 3×10^7^ *L. chagasi* amastigotes. Fifteen days after infection, mice were euthanized with ether and the parasite load was evaluated in Giemsa-stained liver smears and expressed in LDU values (Leishman Donovan units of Stauber  =  number of amastigotes per 600 liver cell nuclei/mg of liver weight) as described [32]. The increase in total body weight and liver/corporal relative weight were also recorded as clinical signs of VL. In order to assess the possible generation of long-term protection 8-week-old female Balb/c mice were vaccinated at weekly intervals, by the sc route, with 3 doses of 100 µg of F1, F2 or F3 recombinant proteins and 100 µg saponin, challenged with 3×10^7^ *L. chagasi* amastigotes on week 4 and euthanized 28 days after infection for evaluation of their liver parasite load.

### Detection of antibodies

Seven days after immunization and 15 days after infection with *Leishmania chagasi*, antibodies of sera were measured in sera by an ELISA assay against NH36 recombinant protein as previously described [33], using 2 µg antigen per well and goat anti-mouse IgG (Sigma) or goat anti-mouse IgG1, IgG2a, IgG2b, IgG3, IgM and IgA horseradish peroxidase conjugated antibodies (Southern, Biotechnology Associates, Birmingham, AL, USA) in a 1∶1000 dilution in blocking buffer. The reaction was developed with *O*-phenyldiamine (Sigma), interrupted with 1 N sulfuric acid, and monitored at 492 ηm. Each individual serum was analyzed in triplicate in double-blind tests. Positive and negative control sera were included in each test. Results were expressed as the mean of the absorbance values (492 ηm) of the 1/100 diluted sera of each animal.

### Antibody-inhibition binding assay

To determine the immunodominance of the sequences predicted to be antibody epitopes of NH36 by the Protean Pad program, the synthetic peptides were obtained and solubilized in DMSO. The FML antigen (2 µg/well) was solubilized in carbonate buffer (pH 9.6), and used to coat flat-bottom 96-well plates for 1 h at 37°C and overnight at 4°C [33]. Plates were washed with blocking buffer and incubated for 1 h at 37°C with a pool of sera of healthy dogs vaccinated with the FML-based licensed vaccine (Leishmune) (n = 10) in the presence or absence of each one of the synthetic peptides diluted in blocking buffer (0.5 to 0.0002 mM). Antibodies were detected using peroxidase-labeled protein-A (Kirkegaard & Perry Laboratories, Gaithersburg, Maryland) at a 1∶16000 dilution, in blocking buffer and the reaction was developed with *O*-phenyldiamine (Sigma), interrupted with 1 N sulfuric acid, and monitored at 492 ηm. The absorbency values of sera pre-incubated with peptides at 0.125 mM was compared to that of total sera with no pre-incubation and expressed as percent of antibody binding inhibition. Sera were analyzed in triplicate. Positive and negative control sera were included in each test.

### Anti-NH36 specific T cell immunity

Seven days after immunization and 15 days after infection with *Leishmania chagasi*, the intradermal response against *L. donovani* lysate (IDR) was measured in the footpads as described earlier [32]. Briefly, mice were injected intradermally, in the right hind footpad, with 10^7^ freeze-thawed stationary phase *Leishmania donovani* promastigotes in 0.1 ml sterile saline solution. The footpad thicknesses were measured with a Mitutoyo apparatus, both before and 0, 24 and 48 h after injection. Injecting each animal with 0.1 ml saline in the left hind footpad served as control. At each measurement, the values of the saline control were subtracted from the reaction due to *Leishmania* antigen. Previous experiments carried out in Balb/c mice and CB hamsters demonstrated that 24 h after inoculation saline treated footpads returned to base levels [32], [34].

All further analyses of cellular immune responses were carried out using 10^6^ splenocytes after 5 days of *in vitro* culturing at 37°C and 5% CO_2_ in RPMI medium and/or 5 µg of recombinant NH36. Flow cytometry analysis (FACS analysis) in a FACScalibur apparatus was performed after splenocyte immunostaining with anti-CD4 (clone GK1.5) or anti-CD8-FITC (clone 53–6.7) monoclonal antibodies (R&D systems, Inc). Secretions of IFN-γ and TNF-α were evaluated in the supernatants of *in vitro* cultured splenocytes with an ELISA assay, using the Biotin Rat anti-mouse IFN-γ (clone XMG1.2), the purified Rat anti-mouse IFN-γ (clone R4-6A2) and the Mouse TNF ELISA Set II kit (BD Bioscience Pharmingen) according to the manufacturer's instructions.

The intracellular production of IFN-γ, TNF-α and IL-10 cytokines by CD4+ and CD8+ T cells was determined using 10 mg/ml brefeldin (SIGMA) for 4 h at 37°C and 5% CO_2_ followed by washing with FACS buffer (2% Fetal Calf serum, 0.1% Na Azide in PBS). Cells were labeled for 20 min. at 4°C in the dark with rat anti-mouse CD4FITC and CD8FITC (R&D systems) in FACS buffer (1/100). After that they were fixed with 4% paraformaldehyde, washed and treated with FACS buffer with 0.5% saponin (SIGMA) for 20 min. at room temperature and then further stained with IFN-γAPC, TNFPE and IL-10PE monoclonal antibodies (BD-Pharmingen), 1/100 diluted in FACS buffer with 0.5% saponin for 20 min., and finally washed and resupended in FACS buffer.

For both the FACS and ICS methods, 30,000 cells were analyzed by flow cytometry on a Becton Dickinson FACScalibur apparatus, and further analyzed using WinMDI (Windows Multiple Document Interface Flow Cytometry Application) Version 2.8 software.

*In vivo* depletion of CD4+ or CD8+ T cells was performed by treating NH36 and F3-vaccinated mice with GK1.5 or 53.6.7 rat IgG MAb on days 2, 4 and 6 before challenge and on day 7 after challenge. Control mice received the NH36sap and F3sap vaccines and 0.05 ml of rat serum ip, equivalent to 0.25 mg of IgG, or nude mice ascitic fluids containing 0.25 mg of anti-CD4+ and/or anti-CD8+ antibodies. As determined by FACS analyses, the efficacy of depletion of CD4+ or CD8+ spleen cells before challenge was of 99.94% or 96% in anti-CD4+ or anti-CD8+ treated mice, respectively. The efficacy of depletion treatment was monitored by the increase in liver parasite load and liver relative weight, 15 days after infection.

### Cross-protective efficacy to infection by *Leishmania amazonensis*

Female Balb/c mice, 8-week-old, were vaccinated at weekly intervals, by the sc route, with 3 doses of 100 µg of NH36, F1, F2 or F3 recombinant proteins and 100 µg of SIGMA saponin [32]. On week 4, mice were challenged in the right hind footpad with 10^5^ *L. amazonensis* (PH 8 strain) metacyclic promastigotes [17] which had been isolated from hamsters and maintained in Schneider's axenic media for 3 successive passages. Measurements of the infected footpad thicknesses were performed weekly with a Mitutoyo apparatus and the thickness values of the non-infected left footpads were substracted from them. The total number of parasites in footpad lesions was determined after sacrifice by Real Time PCR as modified from Manna *et al*., [35] using the primers of *Leishmania chagasi* (Primer forward: 5′GGCGTTCTGCGAAAACCG3′; Primer reverse 5′AAAATGGCATTTTCGGGCC3′ and Probe 5′TGGGTGCAGAAATCCCGTTCA3′) on DNA isolated from promastigotes of *L. amazonensis* (PH 8) and the Taq man system. Briefly, for sample collection, 100 µl of PBS were injected and recovered from each infected footpad. Only 1 µl of each suspension was used for amplification by RTPCR.

### Statistical analysis

The normal distribution of values of each variable was assessed by the Anderson Darling A^2^ test (Analyze-it). Means of normally distributed variables were compared by ANOVA analysis simple factorial test and by one way ANOVA-Tukey's honestly significant difference (Tukey's HSD) post-hoc method (SPSS for Windows). When necessary the confidence interval (95% CI) was also used. Means of non-normally distributed variables were compared by Kruskall Wallis and Mann Whitney non-parametrical tests (Analyze-it). Correlation coefficient analysis was determined on a Pearson bivariate, two tailed test of significance (SPSS for windows).

## Results

**Figure 1:**
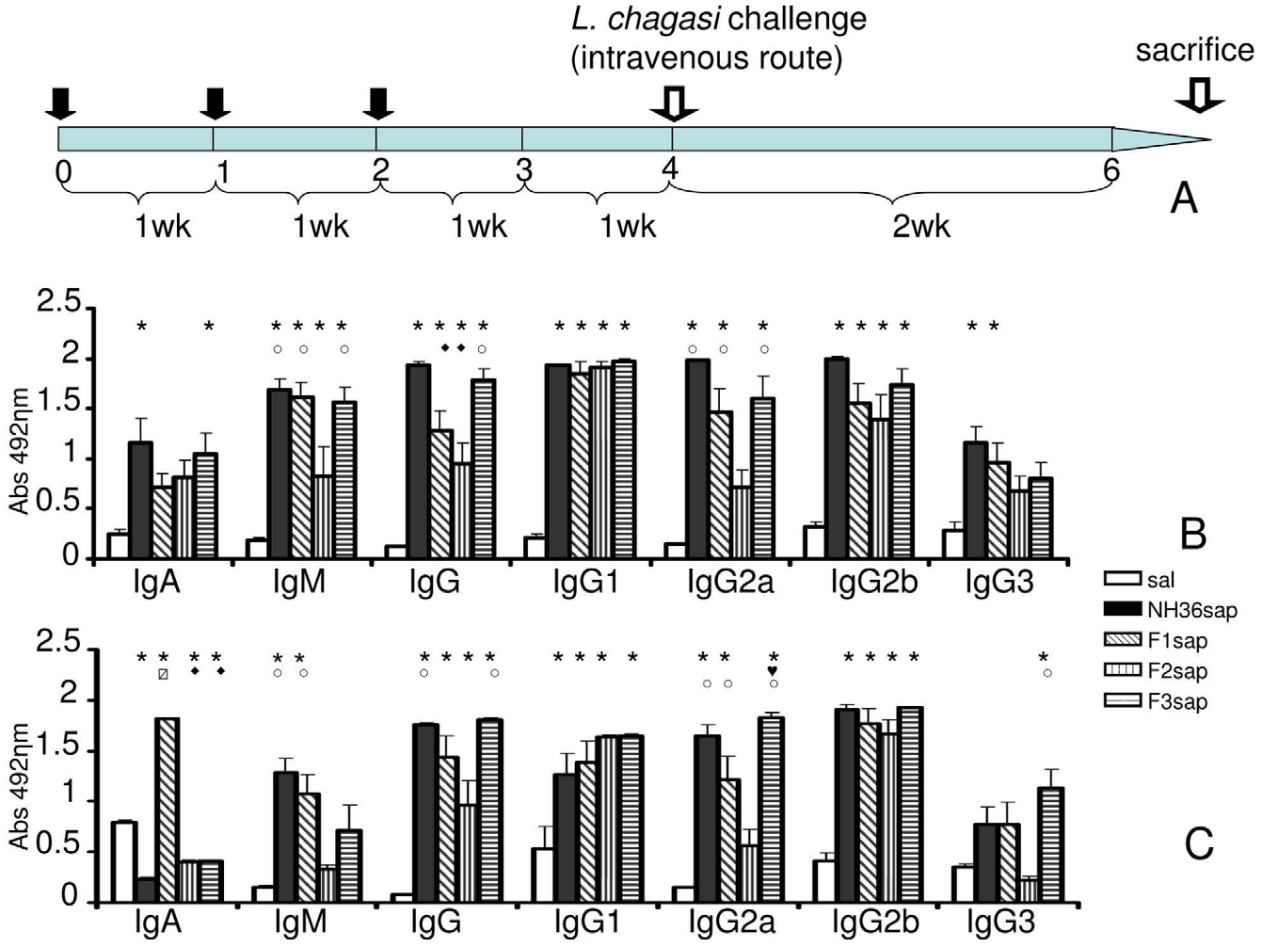
Vaccination, challenge and development of NH36-specific humoral immune response. (**A**) Study design: Balb/c mice were vaccinated with NH36sap, F1sap, F2sap or F3sap at the indicated time intervals, through the sc route, followed by intravenous challenge with *L. chagasi* amastigotes**.** Bars represent the mean ± SE of the absorbance values of anti-NH36 antibodies from 1/100 diluted serum of two independent experiments (n = 11–12 mice per treatment) after immunization (**B**) and after challenge (**C**). * p<0.05 different from the saline control. 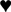 p<0.05 different from F1sap vaccine; **○** p<0.05 different from the F2sap vaccine; ◆ p<0.05 different from NH36sap vaccine; 
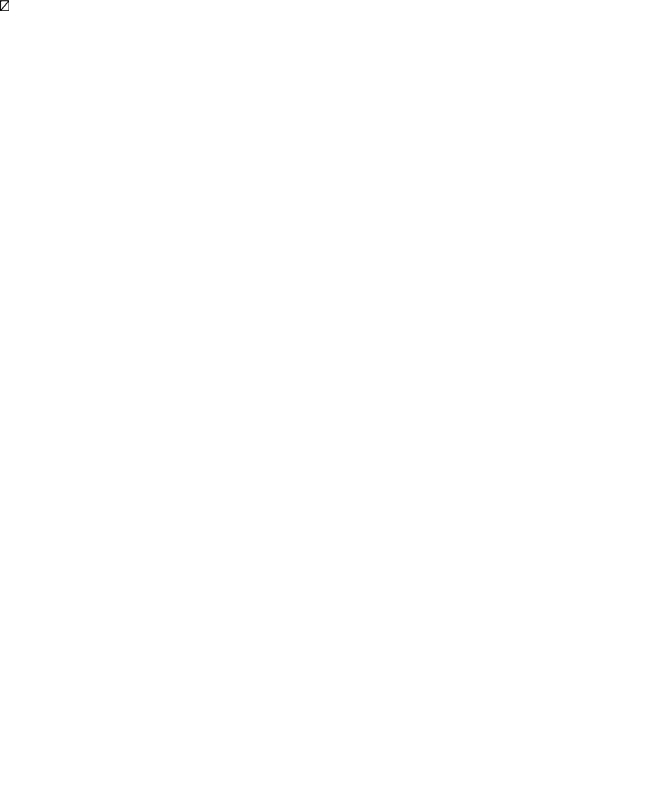
 p<0.05 different from all other vaccines.

**Figure 2:**
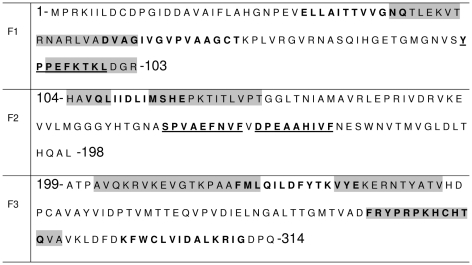
Nucleoside hydrolase NH36 T cell and antibody epitope mapping. The peptide sequence of MHC class II-IA^d^ and IE^d^, haplotype H2^d^ CD4+ T cell epitopes (bold), of MHC class I L^d^-CD8+ T cell predicted epitopes (underlined) and of epitopes for antibodies (grey background) in the F1, F2 and F3 fragments of the NH36 Nucleoside hydrolase of *Leishmania donovani*.

Mice were immunized with NH36, F1, F2 or F3 proteins and saponin (NH36sap; F1sap, F2sap and F3sap vaccines, respectively), challenged with amastigotes of *Leishmania chagasi* on week 4 and euthanized on week 6 (Figure 1A). The humoral response assayed by ELISA disclosed higher antibody levels to NH36 in the sera of vaccinated animals when compared to saline controls after immunization (p<0.004) and after challenge (p = 0.001) (Figures 1B and C). The F3sap vaccine induced IgG levels as high as NH36sap. The IgM and IgG2a levels induced by the F3sap vaccine were as high as the ones elicited by NH36 and F1sap vaccines (Figure 1B). The F2 vaccine induced only IgG2b and IgG1, to the same extent as the other vaccines. After challenge (Figure 1C) only the IgG1 (p = 0.039) and IgM (p = 0.003) responses were lower. The F1sap increased the IgA and IgG and the F3sap, the IgG, IgG2a and IgG3 responses (Figure 1C). The IgG2a level induced by the F3sap vaccine were 70% and 34% higher than those of F2sap and F1sap vaccines, respectively, suggesting that NH36 B cell epitopes for IgG2a antibodies are located mainly in the F3 fragment followed by the F1 fragment. The algorithm program predicted three B-cell epitopes in F3, only one in F1, one between F1 and F2 and one in F2 (Figure 2 and Table 1) and the inhibition of antibody-binding assay was chiefly induced by the synthetic epitopes of F3 (18.82–31.40%) (Table 1) confirming its superiority for the induction of the humoral immune response.

**Table 1:** NH36 antibody epitope mapping.

Fragment	position	Sequence (aa)	antibody binding inhibition (%)
F1	**40**–**57**	N Q T L E K V T R N A R L V A D V A G	0.00
F1–F2	**94**–**108**	P E F K T K L D G R H A V Q L	2.15
F2	**114**–**126**	M S H E P K T I T L V P T	0.46
F3	**202**–**219**	A V Q K R V K E V G T K P A A F M L	31.40
F3	**228**–**239**	V Y E K E R N T Y A T V	18.82
F3	**278**–**291**	F R Y P R P K H C H T Q V A	20.89

Percent of the antibody binding inhibition from sera of Leishmune vaccinated dogs by each of the predicted synthetic peptides in a competitive ELISA assay.

**Figure 3:**
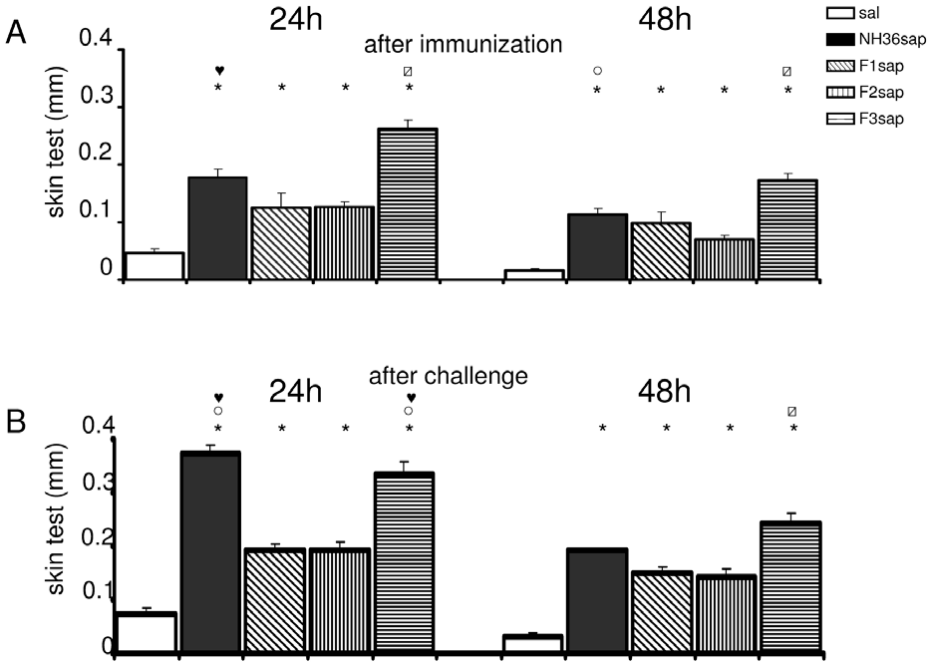
Intradermal response to the leishmanial antigen. (**A**) 24 h and 48 h after complete immunization and (**B**) after challenge with 3×10^7^ amastigotes of *L. chagasi* obtained from hamster spleens. Results of 3 independent experiments with 20–24 mice per treatment (**A**) and 8–11 mice (**B**) per vaccine group are shown as mean + SE. * p<0.05 significantly different from the saline treated controls, 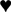 the F1, ○ the F2 or 
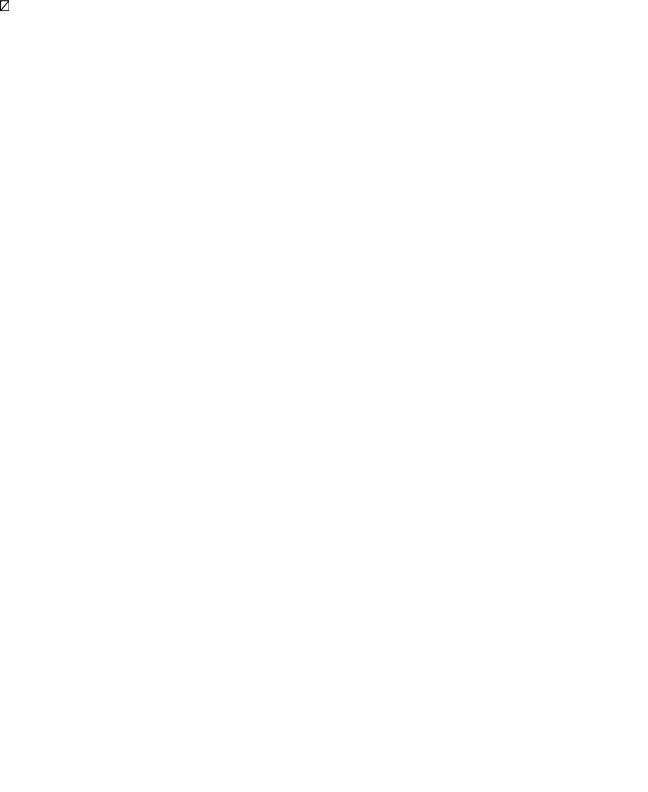
 from all the other vaccines.

The cell-mediated immune response induced by immunization was initially assessed by the IDR for the leishmanial antigen, a strong correlate for protection against human and animal VL that was higher in vaccinated animals than in controls prior to (Figure 3A) and after challenge (Figure 3B) (p<0.0001 in both cases). After immunization, the F3sap vaccine induced the highest footpad swelling (p<0.05), followed by the NH36sap (p<0.05) (Figure 3A). After challenge, the IDR responses were enhanced (p<0.0001) mainly by the NH36sap which was as potent as the F3sap vaccine (p>0.05) at 24 h after injection (Figure 3B). The preponderance of the F3sap vaccine was recovered (p<0.05) 48 h after injection and its best immunogenic properties confirmed (Figure 3B).

**Figure 4:**
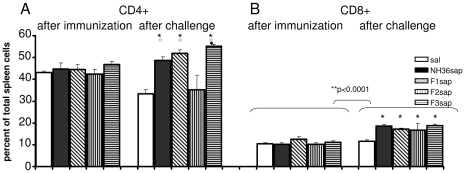
Development of NH36-specific cellular immune response as disclosed by flow cytometry analysis. Splenocytes stained with anti-CD4 (**A**) or and anti-CD8 (**B**) antibodies in vaccinated mice challenged with *L. chagasi*. Results are shown as mean + SE of two independent experiments (n = 14–16 mice per treatment). ** Significant increase in the CD8+ T cell proportions after challenge, * p<0.05 significant differences from the saline treated controls, **○** from the F2sap and ◆ from the NH36sap vaccine.

The proportions of anti-NH36-specific CD4+ and CD8+ lymphocytes in spleens were analyzed by FACS (fluorescence activated cell sorting) (Figure 4A and B). After immunization, the splenic CD4+ T cell proportions (Figure 4A) remained unaltered. After challenge, on the other hand, the F3, F1 and NH36 sap vaccines showed CD4+ T cell proportions increased compared to the saline controls (p<0.05) and the F2sap vaccine (p<0.05). The best performance was achieved by the F3 vaccine with higher proportions of CD4+ T cells than the NH36 vaccine (p<0.05). Of note and as expected due to the progress of VL, after challenge, the CD4+T cell proportions were decreased in saline controls (22%, p<0.05) (Figure 4A). The CD8+ T cell proportions (Figure 4B) that remained unaltered after immunization were, on the other hand, increased by all vaccine treatments after infection (p<0.0001) compared to their respective values before infection (p<0.05) and to the saline control (p<0.05) (Figure 4B).

The levels of cytokines were measured in supernatants of lymphocytes upon *in vitro* stimulation with recombinant NH36 (Table 2). After immunization, and compared to the saline controls (IC95%–1.27 to 9.20 ηg/ml), higher concentrations of IFN-γ were detected only in the NH36sap vaccinated mice (mean = 20.61 ηg/ml). After infection on the other hand, the F1 (mean = 12.35 ηg/ml), F2 (mean = 9.30 ηg/ml) and F3 (mean = 21.84 ηg/ml) vaccines were superior to saline controls (IC95%–1.65 to 8.10 ηg/ml) and the F1sap and F3sap vaccine showed higher IFN-γ levels than the NH36sap vaccine (0.08–11.11 ηg/ml) (Table 2).

**Table 2:** Development of NH36-specific cellular immune response. ELISA of cytokines in supernatants of mice splenocytes.

Treatment		IFN-γ after immunization (ηg/ml)	IFN-γ after challenge (ηg/ml)	TNF-α after immunization (pg/ml)	TNF-α after challenge (pg/ml)
Controls IC95%	Saline	−1.27–9.20	–1.65−8.10	−9.20−87.88	−9.52−188.78
	NH36sap	0.39−40.82	0.08–11.11	8.65–220.89	−10.50–110.38
	F1sap	−1.15–7.81	−3.50–28.21	−0.39–82.80	−38.81–181.67
	F2sap	−5.58–19.00	−3.58–22.19	−12.01–76.98	15.21–117.22
	F3sap	−5.26–19.66	−4.77–48.45	−4.77–148.27	−24.43–250.07
Means of vaccines	NH36sap	**20.61**	5.60	**114.77**	49.94
	F1sap	3.32	**12.35**	41.20	71.43
	F2sap	6.71	**9.30**	32.48	66.21
	F3sap	7.20	**21.84**	71.74	112.82

The mean of one group of data of vaccinated mice (results of one experiment with n = 7–8 mice per treatment) is or not included in the 95% CI of control groups.

As detected for IFN-γ, the TNF-α levels after immunization (Table 2) only increased in the supernatants of NH36sap treated mice (mean = 114.77 pg/ml) when compared to the saline injected controls (IC95%–9.20 to 87.88 ηg/ml). After infection, the TNF-α secretion which correlates to the IFN-γ secretion (p<0.0001) also showed the highest values in the F3sap and F1sap vaccinated individuals (447.44 pg/ml and 431.40 pg/ml, respectively, not shown). This experiment was the only one in the whole investigation in which neither the ANOVA-Tukey's HSD nor the Kruskall Wallis-Mann Whitney tests disclosed any significant differences. For this reason we used the IC95% for analysis.

**Figure 5:**
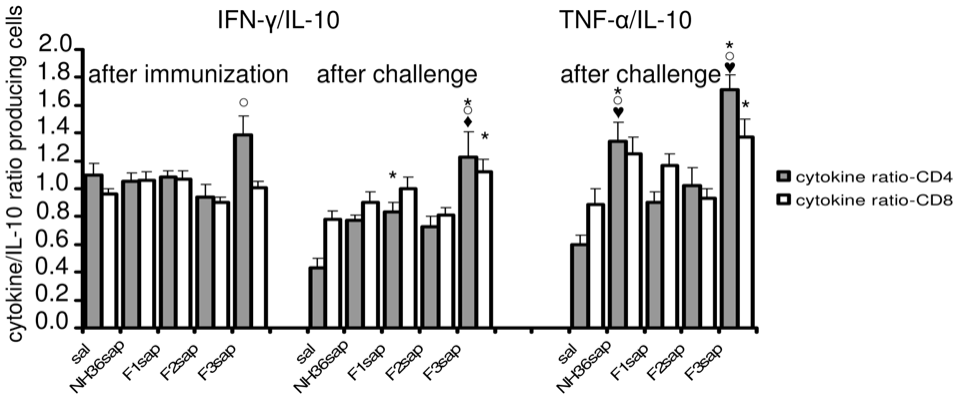
Development of NH36-specific cellular immune response as disclosed by intracellular staining analysis of splenocytes *in vitro* cultured with NH36 before and after *L. chagasi* infection. Anti-CD4-FITC and anti-CD8-FITC antibodies were used for labeling the cell surfaces and anti-IFN-γ-APC, anti-TNF-α-PE and anti-IL-10-PE for intracellular staining. In order characterize the TH1 response bars represent the ratio of IFN-γ/IL-10 and TNF-α/IL-10 producing cells. Results represent mean + SE of two independent experiments (n = 7–8 mice per treatment). * p<0.05 indicate significant differences from the saline treated controls, 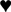 from F1sap, ○ from F2sap, and ◆ from the NH36sap vaccine.

The expressions of IFN-γ, TNF-α and IL-10 were also studied by the ICS (intracellular cytokine staining) approach. In order to characterize the potential TH1 response generated by vaccination with the NH36 peptides we show the results as ratios of IFN-γ/IL-10 and TNF-α/IL-10 CD4+ and CD8+ producing cells (Figure 5). Our analysis disclosed the predominance of the F3 domain of the Nucleoside hydrolase which induced the highest TH1 response after immunization, which was sustained after challenge. Indeed, after immunization, the ratios of IFN-γ/IL-10 CD4 producing cells increased significantly (p<0.009) mainly in F3sap vaccinated mice compared to those of mice vaccinated with F2sap (p<0.05) while no differences were found in CD8+ T cells. After challenge, the ratios of IFN-γ/IL-10 CD4+ T cells also showed significant increases (p<0.0001). The F3sap vaccinated mice showed a 65% increase compared to saline controls and enhancements compared to all other vaccines (p<0.05) except for the F1sap which itself showed a 32% increase over saline controls (p<0.05). The CD8+ IFN-γ/IL-10 producing cells also showed differences (p<0.031) mainly due to the increase in F3 vaccinated mice (p<0.05) (Figure 5).

Furthermore, the TNF-α/IL-10 CD4+ response after challenge was stronger than the IFN-γ/IL-10 CD4+ response (p<0.0001). Indeed, the TNF-α/IL-10 CD4+ ratios of the F3sap vaccinated mice showed a 29% increase compared to the IFN-γ/IL-10 CD4+ ratios for the same group (p<0.0001). Also, different from what detected for IFN-γ, both the NH36 and the F3sap vaccinated mice showed TNF-α/IL-10 CD4+ ratios higher than those of saline controls, F2 and F1 vaccinated animals (p<0.05). Additionally the F3 vaccine ratio was 22% greater than that of the NH36 vaccine (p<0.05) (Figure 5). Finally, the TNF-α/IL-10 CD8+ producing cells were only increased in the F3 vaccinated mice (p<0.05). Our results indicate that the response induced by the F3 peptide (C-terminal domain) overcomes the one induced by the cognate NH36 protein suggesting that it holds the main NH36 sequences responsible for the TH1 immune response. The TNF-α/IL-10 ratio also suggests, in the F3 sequence, the presence of more epitopes interacting with CD4+ than with CD8+ T cells (the mean of CD4 = 1.71 falls outside the IC95% of CD8 = 1.12–1.62). This is not the case for the NH36sap vaccine which stimulates similar proportions of both subsets of T cells (the mean of CD4 = 1.3 is included in the IC95% of CD8 = 1.02–1.54).

**Figure 6:**
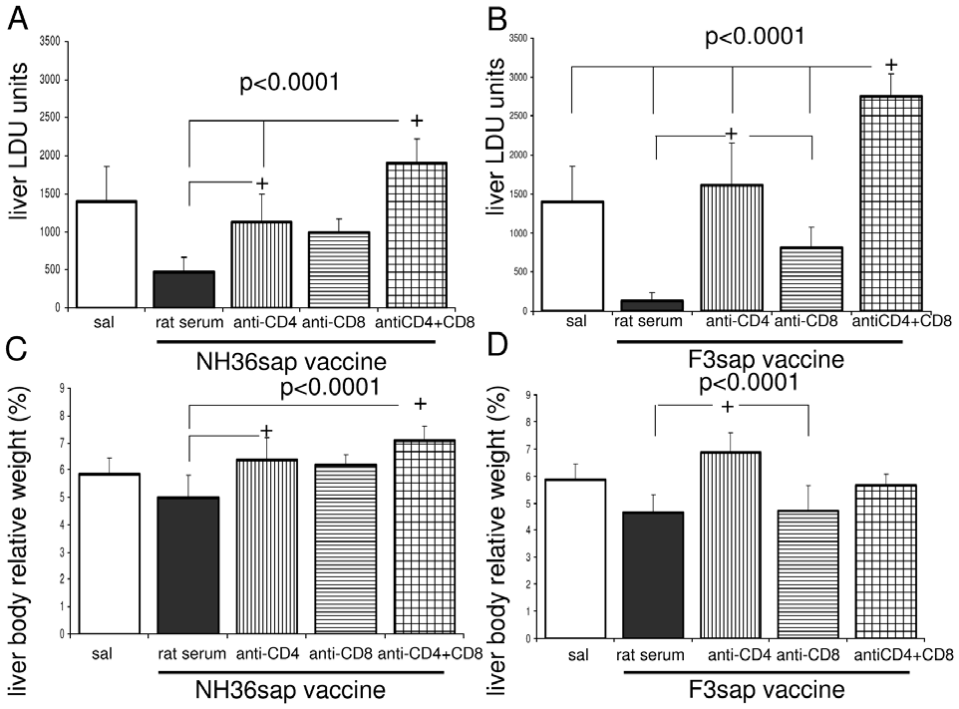
Development of cell-mediated immune response as disclosed by *in vivo* depletion with anti-CD4+ and anti-CD8+ monoclonal antibodies. *Leishmania chagasi* parasite-load (**A** and **B**) and percent of liver/corporal relative weight (**C** and **D**) in mice vaccinated with NH36sap and F3 sap vaccine and treated with rat serum, anti-CD4+ or anti-CD8+ or the combination of anti-CD4+ and anti-CD8+ MAbs. Maximal parasite load reduction was achieved in mice that received either the NH36sap or the F3sap vaccines and rat serum (rat IgG) as controls for antibody treatment. Bars represent the mean + SD (5 mice per each treatment). The parasite load is expressed in LDU values (number of amastigotes per 600 liver cell nuclei/mg of liver weight) (*A* and *B*). Hepatomegaly was assessed by the individual increment in liver relative weight expressed as percent of the body weight. *+* p<0.05 significant differences between treatments.

The *in vivo* depletion assay with anti-CD4+ and anti-CD8+ monoclonal antibodies (Figure 6) on mice immunized with NH36sap and F3sap vaccines confirmed the results of ICS. When compared to the saline control (mean = 1402.9 LDU) a 90.5% reduction was obtained with the F3sap vaccine (mean = 132.56 LDU) (Figure 6B) while only 65% was obtained after vaccination with the NH36 sap vaccine (mean = 478.95 LDU) (p<0.05) (Figure 6A), indicating that the F3sap vaccine induced a 25.2% increase in protective efficacy against mice VL.

In correlation to what was detected for the TNF-α/IL-10 ratios after infection (Figure 5), in NH36sap vaccinated mice, the anti-CD4+ treatment induced 59.5%, and the anti-CD8+, 52% of the total LDU counts of mice treated with both antibodies simultaneously, indicating a similar degree of contribution of CD4+ and CD8+ T cells (Figure 6A *;* p>0.05) to the vaccine induced protection. Also in correlation with the results of TNF-α/IL-10 ratios (Figure 5), protection due to the F3sap vaccine was mainly mediated by the CD4+ T cells (p<0.05) with a lesser contribution by CD8+ cells, since treatment with anti-CD4+ or antiCD8+ antibodies led to increases in susceptibility of 59.0% and 29.5%, respectively (Figure 6B). Coincidentally, enhanced liver/body relative weight (hepatomegaly), was promoted in NH36 vaccinated mice treated with anti-CD4+ MAb or anti-CD4+ plus anti CD8 + Mabs together (Figure 6C) and in the F3sap vaccinated mice treated with anti-CD4+ antibodies alone (Figure 6D). These results confirm that while the NH36sap global protection is mediated by CD4+ and CD8+ lymphocytes, the contribution to immune response of the F3 protein is mainly mediated by CD4+ T cells with a minor contribution of CD8+ T cells.

**Figure 7:**
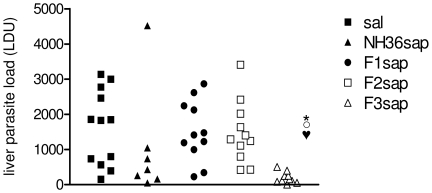
Protective efficacy of vaccinated mice against *L. chagasi* infection. The individual *L. chagasi* liver parasite load of vaccinated and control groups is expressed in LDU values (number of amastigotes per 600 liver cell nuclei/mg of liver weight) of 2 independent experiments, each with 4–8 mice per vaccine group. *p<0.05 significant differences from the saline controls, 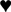 from the F1sap and ○ from the F2sap vaccines. The mean averages of LDU values are: 1632.64 (sal); 1027.50 (NH36sap); 1806.49 (F1sap); 1469.91 (F2sap) and 192.14 (F3sap*)*.

Regarding the parasitological assessment of infection and as expected from the results of the humoral and cellular immune responses, significant differences were found (p = 0.011) and the F3 vaccine induced the highest efficacy with a 88.23% parasite load reduction (p<0.05) (Figure 7). The reduction due to F3 vaccine was not significantly different from that due to the NH36 vaccine (37.06%), which in spite of that, exhibited more than 1000 LDU in 2 of 6 vaccinated mice (Figure 7). The F3sap vaccine also induced a 20.9% reduction (p<0.05) of the liver/body relative weight (not shown). The F1 vaccine, on the other hand, did not provide protection (Figure 7) in spite of the results of the antibody, FACS, ICS and cytokine analyses.

Epitope prediction programs disclosed three H2-Ld peptide nonamers for CD8+ lymphocytes in NH36 (Figure 2). The Y**P**PEFKTK**L** CD8+ epitope is located in F1 fragment and the SPVAEFNVF and DPEAAHIVF epitopes in the F2 fragment. Among the epitopes for CD4+ lymphocytes, the peptides ELLAITTVVGNQ (IA^d^ allele) and FRYPRPKHCHTQ (IE^d^ allele) with the highest predicted affinity, are located in F1 and F3, respectively (Figure 2) while two peptides with lower affinity are located in F3, one in F1 and one in F2.

**Figure 8:**
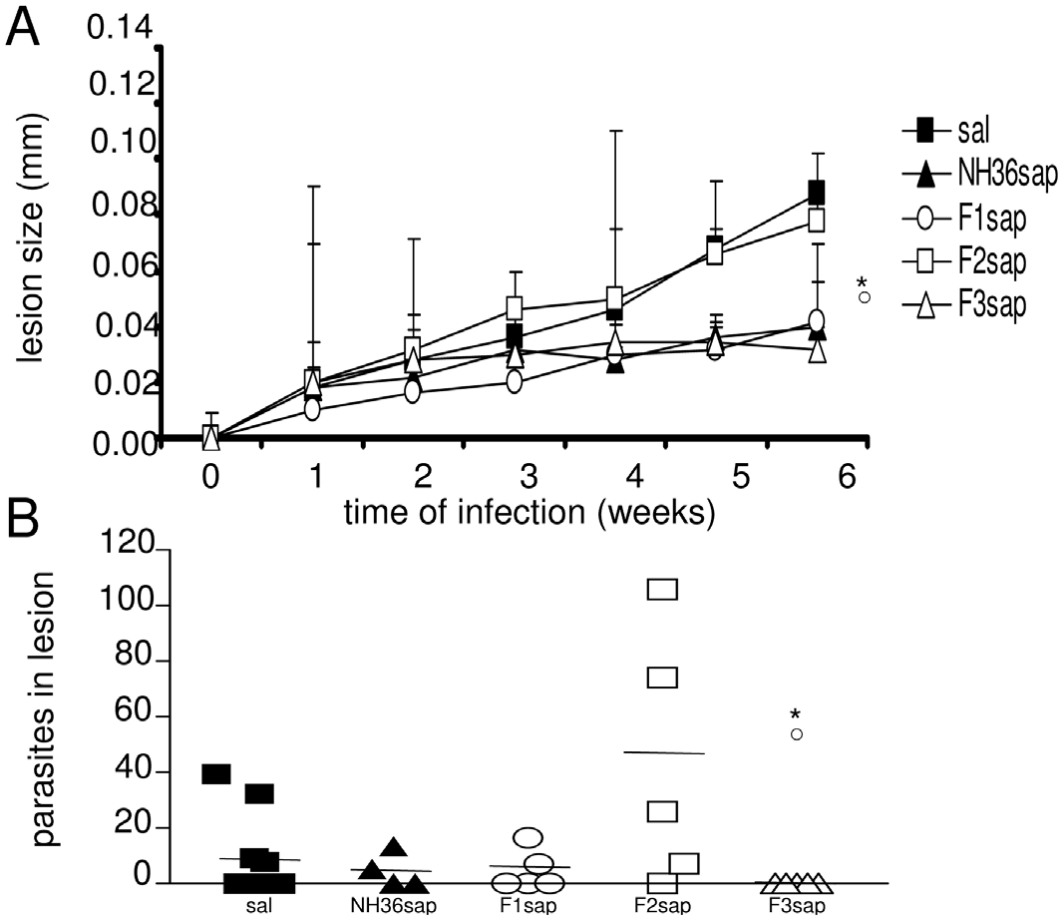
Protective efficacy of vaccinated mice against *L. amazonensis* infection. (**A**) Evolution of the size of footpad lesions of mice challenged with 10^5^ metacyclic promastigotes of *L.amazonensis* one week after completing vaccinations. Results are from 2 independent experiments with 5 animals per treatment. Lesions development was followed by measuring the increment in the thickness of the infected footpad compared to the thickness of the contra-lateral non-infected footpad. Results represent the mean + SE of the footpad measurements. (**B**) The number of promastigotes of *Leishmania amazonensis* in the footpad lesions as disclosed by Real Time PCR assay. The horizontal lines represent the mean averages. *p<0.05 significant differences from the saline controls and ○ from the F2sap vaccines.

Aiming to identify the NH36 domain responsible for the NH36 cross-protection to other infections caused by *Leishmania* species [14], [16], [17], we also assayed the protective efficacy of NH36 and its fragments in the TL model. Vaccinated mice were challenged with infective *L amazonesis* promastigotes on their footpads. Significant differences in footpad sizes were detected until week 6 after infection (p<0.0001) (Figure 8A). The NH36sap, F1sap and F3sap vaccines reduced lesion sizes in comparison to the F2sap vaccine (p<0.05) and to the saline treated controls (p<0.05). Furthermore, the parasite load evaluation in footpad lesions coming from *Leishmania amazonensis* DNA dosage by Real Time PCR performed on week 6 after infection, disclosed, in agreement to what has already been described for protection against *L. chagasi* (Figures 6 and 7) that only the F3 fragment (C-terminal domain) was effective against *L.amazonensis* infection (Figure 8B). Indeed, significant differences were found in the parasite load (p = 0.039). Despite the spontaneous negativation of 5 of the 10 untreated controls, the F3sap vaccine significantly reduced the parasite load to zero in all mice promoting 100% reduction of parasite load (p<0.05) when compared to the untreated controls (mean = 9.87 promastigotes) and to the F2 vaccinated mice (mean = 42.6 promastigotes). In both the VL and TL models, the C-terminal domain of NH36 (F3) is the main target of the immunity and protective efficacy against the pathogen infection with a minor immunogenic contribution detected in the N-terminal domain (F1).

Although in some variables (anti-NH36 antibodies, ratio of TNF-α/IL-10 CD4 producing cells, footpad swelling in vaccination against *L. amazonensis*) no significant differences were found between the effects of the NH36 and the F3sap vaccines, in many others, the superiority of the F3 over the NH36 vaccine was evident. We calculated the increment in the immunoprotective effect of the F3 vaccine taking in consideration all the variables that showed significant differences between the two formulations (Table 3). We found that the F3 vaccine developed a 36.73% higher average protective effect than the NH36 vaccine.

**Table 3:** Superiority of the F3 over the NH36 vaccine.

Variable	F3	NH36	Enrichment
IDR 24 h after immunization	0.262	0.178	32.06%
IDR 48 h after immunization	0.173	0.114	34.10%
IDR 48 h after challenge	0.243	0.191	21.40%
Ratio IFN-γ/IL-10 CD4 T cells	1.23	0.77	37.39%
Reduction of parasite load (*in vivo* depletion)	90.5	65.90	27.18%
Reduction of parasite load *L chagasi*	88.23	37.06	57.99%
Reduction of parasite load *L. amazonensis*	100	53.00	47.00%
Mean + SD			36.73+12.33%

Calculation was performed according the following equation  =  (F3-NH36/F3) values x 100 =  protective effect increment.

**Figure 9:**
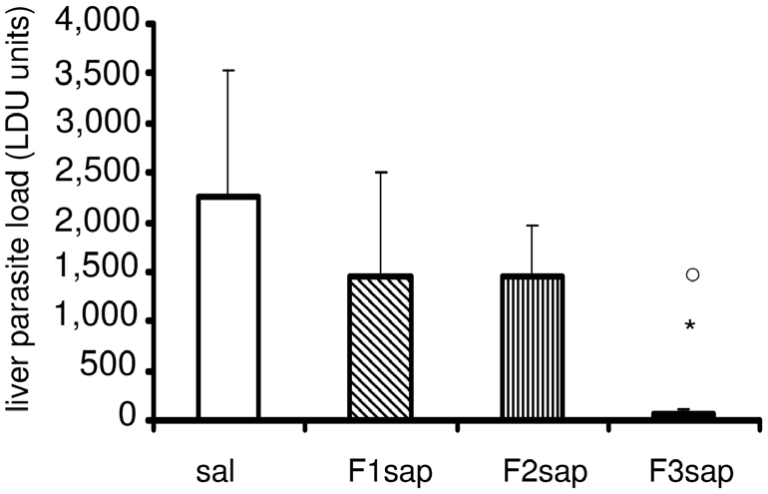
Long-term protection generated by the F3sap vaccine. Balb/c mice were vaccinated with NH36sap, F1sap, F2sap or F3sap at the indicated time intervals, through the sc route, followed by the intravenous challenge with *L. chagasi* amastigotes 28 days after the last immunization**.** Bars represent the mean ± SD of the individual parasite load in liver measured by LDU (one experiment, n = 3–4 mice). *p<0.05 significant differences from the saline controls and ○ from the F2sap vaccine.

Furthermore, the possible long-term protection generated by the F3sap vaccine was assayed in Balb/c mice submitted to 3 weekly interval vaccinations with either F1, F2 or F3 peptides in saponin formulations and challenged, 4 weeks after completing all vaccinations. The results of parasite load evaluation are summarized in Figure 9 and disclosed a 97.5% level of protection generated only by the F3 vaccine compared to the saline controls (p<0.05) and the F2sap vaccine (p<0.01) revealing that the C-terminal domain of NH36 includes the epitopes of the Nucleoside hydrolase NH36 involved in the induction of long-term protection against VL.

## Discussion

Our study has disclosed very reliable information about immunoprotection against VL. As expected for the protection generation, significant inverse correlations were found between the decrease of both liver LDU and liver/body relative weight and the increases of IDR (−p = 0.049) and ratios of TNF-α/IL-10 CD4+ producing cells (−p = 0.014). Accordingly, we demonstrated that the F3 peptide vaccine was capable of increasing the IDR and the ratios of TNF-α/IL-10 CD4+ T cells and of decreasing the parasite load and hepatomegaly. Thus, in our model and confirming previous results [36]–[43], the increase of IDR and the ratio of TNF-α-CD4+ producing cells are the immunological parameters correlated with protection against VL. Also similar to what has already been reported [44], [45] the ratios of IFN-γ/IL-10 producing CD4 T cells were not correlated to the decrease of LDU or hepatomegaly (p>0.05), but were correlated to the increase of IDR, CD4+ T cells and IgG, IgG2a and IgG2b antibodies (p<0.023 for all variables). Therefore, in our model, the increase in IFN-γ producing cells was not a correlate for protection and the F1 vaccine which indeed promoted these increases did not give protection.

The epitope prediction programs disclosed the CD8+ epitope Y**P**PEFKTK**L** in F1 and the SPVAEFNVF and DPEAAHIVF CD8+ epitopes in F2 while no CD8+ epitope was found in the F3 fragment. In agreement to that, the ICS and *in vivo* depletion assays disclosed that protection generated by the F3 vaccine is predominantly mediated by CD4+ T cells, suggesting that the CD8+ stimulating activity of the NH36 vaccine is related to those epitopes located in the F1 and/or F2 sequences. Furthermore, the epitopes with the highest predicted affinity for CD4+ lymphocytes, are located in F1 and F3, while the two lower affinity CD4 peptides are located in F3. Epitope prediction therefore, suggested the strongest capability of F3 for CD4+ T cell mediated protection and antibody synthesis.

After challenge, and as described before [28] increased levels of IFN-γ and TNF-α were secreted by mice vaccinated with F1 and F3 peptides but not with the NH36 protein. Our results correlate to the presence of 3 important CD4+ T cell epitopes in F3. They correspond to a sequence of 14, 12 and 14 amino acids, respectively, making a 40 amino acid potent sequence. For each vaccine dose containing NH36 (314 amino acids) these 40 amino acids represent only 12.7% of the main active component. On the other hand, they represent a 34.8% of the 115 amino acid sequence of peptide F3 meaning that the F3 vaccine has a 2.7 enrichment of the main active component. This might explain the earlier induction of IFN-γ and TNF-α by F3sap vaccine, its strongest efficacy (88–90.55% of reduction of parasite load) and the lower potential of the NH36 vaccine for generation of DTH, IFN-γ/IL-10 ratio of CD4+ and CD8+ T cells as well as the reduction of hepatomegaly and parasite load detected in this (37.06% and 65.90%) and in previous investigations (67.80–79.00%), respectively [14], [25].

There is an increase also in the ratio of TNFα/IL-10 but not of IFN-γ/IL-10 CD4+ producing cells in NH36 vaccinated mice after challenge that was not detected by the cytokine ELISA assay. This might be due to the higher sensitivity of the ICS technique. Another possible reason for that would be the sequence of events involved in the CD4+ T cell differentiation [46]. TNF-α is considered to be the most ubiquitous cytokine and it is produced by most activated CD4+ T cells [reviewed in 46] generated under conditions that favor TH1-cell differentiation. It proved to be important in protection against VL [36]–[43]. Optimal protection would be achieved by having a population of multifunctional T cells that can mediate an effector function quickly and have a reservoir of memory T cells that secrete IL-2, TNF-α or both. Once CD4+ T cells have developed into IFN^+^-TNF^+^-IL-2^+^ T cells they have three potential fates: they can persist as memory or effector T cells, they can further differentiate into less functional T cells or they can die following activation [46].

The model for effector and memory CD4+ T-cell differentiation of Seder [46] involves the earliest secretion of TNF-α followed by IL-2, by TNF-α and IL-2 and by the later IFN-γ-TNF-α-IL-2 secretion in CD4+ cells that can persist as memory or effector T cells. The finding of TNF-α producing cells in mice vaccinated with NH36sap and challenged could indicate the existence of an early effector cell-response generated by the vaccine. On the other hand, mice vaccinated with the F3 peptide that contains a higher density of the immunoprotective epitopes show a more advanced stage of CD4+ T cell differentiation with a more intense and suggestive combined secretion of IFN-γ and TNF-α that indicates the optimized effector function of CD4 T cells and the potential generation of long-term memory T cells. Therefore, our results might indicate the presence of an early TNF-α secreting response by CD4+ T cells of mice vaccinated with the less potent NH36 vaccine and the presence of single-TNF-α and/or double TNF-α-IFN-γ producers in mice vaccinated with the most potent F3 fragment. The percents of cell producing IFN-γ or potential double TNF-α-IFN-γ producers are much lower in NH36 mice and yet not significantly different from the saline control. In agreement with the results of ICS, the ELISA assay of splenocyte supernatants after infection, which probably correspond to multiple different cells, shows that the F3, not the NH36, induced an increase in IFN-γ secretion. In our study the ICS was carried out by the independent labeling of the T cell populations secreting IFN-γ, TNF-α and IL-10 and not by multiparameter cytometry. In order to establish if the increase in TNF-α and IFN-γ producing T cells caused by the F3 vaccination treatment is due to TH1 multifunctional T cell differentiation [46], [47] or if it is the result of the secretion by distinct independent T cells, a further multiparameter cytometry analysis will be necessary. Our preliminary experimental results of mice challenged one month after vaccination however suggest the existence of memory T cells and the induction of long-term protection by F3 vaccine. Indeed, 97.5% of parasite load reduction was detected disclosing that the vaccine is able to generate both effector and memory T cells responsible for the immunoprotective response. Further experiments with challenges performed after one month of complete vaccination should bring relevant information on the extension of the long-term protection generated by the F3 fragment.

Vaccines eliciting a high frequency of single-positive IFN-γ producing cells may be limited in their ability to provide durable protection [46], [47]. Most vaccine studies for infections requiring TH1 responses measure the frequency of IFN-γ producing cells as the primary immune correlate of protection. Although IFN-γ is clearly necessary, using it as a single immune parameter may not always be sufficient to predict protection [47]. TNF-α is another effector cytokine that can mediate control of intracellular infections. Indeed, IFN-γ and TNF-α synergize in their capacity to mediate killing of pathogens [47]. As described in our investigation for the F3 vaccine mediated protection against *L. chagasi*, Darrah et al. [47] reported that vaccine-elicited protection against *L. major* was completely abrogated upon depletion of CD4+ T cells. Also, depletion of IFN-γ or TNF-α at the time of infection abolished vaccine mediated protection [47]. The total frequency of antigen-specific IFN-γ+ cells was not predictive of vaccine-elicited protection. In contrast, the analysis showed a correlation between the frequency of multifunctional (IFN-γ, IL-2 and TNF-α triple-positive) CD4+ T cells and the degree of protection [47].

In our investigation, the analysis of the cell-mediated immune response confirmed the epitope prediction analysis indicating that protection induced by NH36 vaccine is mediated by equal proportions of CD4+ and CD8+ T cells and it is even extended by protection generated by F3 vaccine which is mediated predominantly by CD4+ with a minor contribution by CD8+ T cells. This is an outstanding property of the C-terminal domain of NH36 considering the difficulties to obtain CD4+ mediated immune protection against protozoa infections [30]. The CD8+ T cell contribution of the NH36 vaccine might be related to the CD8+ epitopes predicted for the F1 sequence.

NH36 is a strong phylogenetic marker for the *Leishmania* genus [1], [2] and the finding of 93–99% of homology between the NHs amino acid sequences of *L. donovani, L. major* [7], *L. chagasi*, *L. infantum*, *L. mexicana, L. amazonensis* and *L. tropica* [7], [13] explains the previously detected cross-protection [14], [16], [17]. Vaccination with NH36 of *L. donovani* promoted an 88% reduction of the *L. chagasi* parasite load [14] and induced a 65%, 80.4% and 97% reduction of the skin lesion sizes or parasite loads of mice with tegumentary leishmaniasis by *L. mexicana* [14], *L. amazonensis* [16] and *L. major* [17], respectively. We showed that while reduction of the lesion size due to *L. amazonensis* infection was promoted by immunization with either the F1 or F3 of NH36, in agreement with our findings on modulation of infection by *L. chagasi,* the reduction of parasite load was only determined by the F3sap. The increase in size of infected footpads is a specific measure of the progress of infection since the normal increase of footpad size with corporal growth is subtracted. It might be argued that the lesion size might also be influenced by the amount and nature of the local inflammatory response [48] which might be mediated by the B2R receptor for the released bradykinin at the local of infection [49]. In Swiss and C57BL/6 mice infected with *L. amazonensis*, the histopatological primary footpad lesion analysis showed liquefactive necrosis and inflammatory infiltrate mainly consisting of macrophages filled with amastigotes and rare lymphocytes [48]. Interestingly, the study of the dermal ear infection with *L. amazonensis* in C57Bl/6 mice showed that the absence of the TLR2 receptor determined the reduction of both the parasite load and the recruitment of inflammatory cells [50]. On the other hand, the generation of an inflammatory response is expected to determine a bradykinin-mediated partial protection of mice vaccinated against leishmaniasis using a saponin adjuvant [Nico D, Souza LOP, de Almeida LN, Monteiro ACS, Scharfstein J, et al., unpublished results]. In spite of those evidences, in the present investigation, the footpad sizes were significantly diminished only by vaccination treatment with NH36, F1 and F3 and saponin but not with F2-saponin or saline indicating that the sustained small footpad sizes are more related to the protection generated towards the antigenic sequences used for vaccination than to the inflammatory response generated by the *Leishmania* infective challenge or by the saponin adjuvant.

In this investigation, the RTPCR although sensitive enough for dog diagnosis [35] generated results that were not directly related to the increase of the whole footpad lesions (Pearson correlation coefficient, p = 0.726). While all control animals developed footpad lesions (mean+ SE = 0.008+0.0036) only 5 out of 10 showed parasites. In our model, the footpad swelling detected the protective contribution of the F1 and F3 vaccines while the RTPCR disclosed only the most potent F3 peptide as the main domain of NH36 involved in generation of immunoprotection. Both results were significantly different from controls and are in agreement with the results obtained in the vaccination against *L. chagasi* infection confirming the C-domain of NH36 as the one containing the more important epitopes of potential cross-protection. The finding of few parasites in footpads might be related to the lower infective challenge used in this work. We used 10^5^ infective promastigote of *L. amazonensis,* as Al Wabel et al. did for Balb/c vaccination with recombinant NH36 against *L. major* infection [17]. Coelho et al. [51], on the other hand, used 10^6^ *L. amazonensis* infective promastigotes and obtained more enhanced increases of Balb/c footpads. The use of a higher inoculum would probably also determine an increase of the parasite load in our model. However, it is worth noting that in our investigation, although using a lower challenge, significant differences concerning protection were found.

Similar to what described by Kao et al. for the *P. aeruginosa* Type IV pilus vaccine [29] the superiority of the F3 vaccine over the NH36 cognate protein vaccine is evident by the 36.73% average increase in IDR, IFN-γ/IL-10 CD4 T cells and reduction of *L. chagasi* and *L. amazonensis* parasite load. This is probably related to the 2.7 enrichment in the important epitope sequences which represent 34% of the F3 peptide. Vaccine protection could be further improved by the generation of shorter recombinant peptides of the F3 fragment composed only of the combination of the most relevant epitopes (research in progress).

The F1sap vaccine induced lower levels of antibody response that was not inhibited by the synthetic predicted peptides. The F2 vaccine, on the other hand, showed the worst performance of the three fragments tested. It showed the lowest inhibition of binding assay and was only able to increase the anti-NH36 IgG2b and IgG1 antibodies to the same extent as the other vaccines. This increase seems to be related to specific properties of the QS21 saponin-containing adjuvant [52] since no other antibody enhancements were detected after vaccination with F2. After challenge, the levels of IgG1 antibodies were reduced in vaccinated mice but increased in controls indicating that protection against the VL-TH2 expansion was achieved by all vaccines despite the typical TH1/TH2 mixed response expected for the use of saponin adjuvant [52]. The IgM response was also reduced after challenge confirming the establishment of a secondary IgG antibody immune response.

Despite the many antigens tested for vaccination in laboratory models [53] only two other formulations are under analysis as tentative synthetic vaccines against *Leishmania* [54], [55]. Thirty overlapping 9-mer peptides of the kmp-11 protein of *L. donovani* trigger IFN-γ secretion by human CD8+ T cells and contain many potential HLA class I-restricted T cell epitopes that can be presented by different HLA molecules [54]. The other formulation is the polyprotein Leish110f composed of the TSA, LmSTI1 and LeIF candidates fused in tandem induced mice protection mediated by CD4+ T cells with a higher secretion of TNF-α followed by IFN-γ and IL-2 [55].

To our knowledge the description of the C-terminal domain of the NH36 antigen as the main active component in protection against leishmaniasis constitutes the first case of a licensed vaccine to evolve to a DNA, to a recombinant defined protein formulation and then progress to a synthetic vaccine. Our findings contribute to the potential development of synthetic vaccine formulations against parasites of the *Leishmania* genus and against multiple microorganisms which have NHs in their replication pathways.
